# Perspectives on ADHD in children and adolescents as a social construct amidst rising prevalence of diagnosis and medication use

**DOI:** 10.3389/fpsyt.2023.1289157

**Published:** 2024-01-05

**Authors:** Tobias Banaschewski, Alexander Häge, Sarah Hohmann, Konstantin Mechler

**Affiliations:** ^1^Department of Child and Adolescent Psychiatry and Psychotherapy, Central Institute of Mental Health, Medical Faculty Mannheim, Heidelberg University, Mannheim, Germany; ^2^Department of Child and Adolescent Psychiatry and Psychotherapy, University Medical Center Hamburg-Eppendorf, Hamburg, Germany

**Keywords:** ADHD, attention-deficit/hyperactivity disorder, prevalence, overdiagnosis, treatment, stigma

## Abstract

The diagnosis of attention-deficit hyperactivity disorder (ADHD) is based on the presence of pervasive, persistent symptoms of inattention and/or hyperactivity/impulsivity typically emerging early in life and resulting in significant functional impairment. In contrast to a worldwide epidemiological prevalence of approximately 5% in children and 2–3% in adults, there are significant variations in the prevalence of administrative ADHD diagnoses and medication use. We assert that in order to explore the underlying dynamics of this phenomenon, a thorough understanding of the construct ADHD is necessary. We contend that ADHD is not a natural entity that unfolds within an individual and can be understood independent from societal and environmental factors, but rather that ADHD as a diagnosis can better be conceptualized as a valid and pragmatically useful social construct. Decisions to diagnose and treat ADHD should follow a person-centered approach and be focused on functional impairment within a socially constructed, context-dependent and environmentally contingent model.

## 1 Introduction

The concept of attention-deficit hyperactivity disorder (ADHD), like the concepts of other psychiatric disorders, has undergone refinement and development over the past five decades, to its current inclusion in DSM-5 (1) and ICD-11 (2).

First clinical descriptions of children with problems of focus, hyperactivity and lack of impulse control appeared around the time of enlightenment and industrialization, when most governments in Western Europe and the US adopted policies of compulsory education, turning most children from workers into students (3). It has been argued [e.g., (4)] that despite the undeniable neurobiological basis of ADHD, the problematic patterns of behavior are substantially uncovered in “classroom”-settings with a special focus on conformity and learning. As the importance of academic performance (especially in the face of a growing competition for jobs and prosperity in a globalized world) has significantly increased over the last decades (or even centuries), this “push for performance” might explain and predict the increasing prevalence of ADHD diagnoses (4, 5).

The current categorical diagnosis of ADHD is based on the presence of pervasive, persistent symptoms of inattention and/or hyperactivity/impulsivity typically emerging early in life and resulting in significant functional impairment. A crucial criterion involves the presence of symptoms across multiple settings (e.g., home and school) and their consequential impact on academic, social, or occupational functioning.

ADHD is a common disorder with a worldwide epidemiological prevalence of approximately 5% in children (6–8) and 2–3% in adults (9). A multitude of epidemiological and clinical studies point to the common co-occurrence of ADHD with other psychiatric disorders, including depression, anxiety disorders, dyslexia, autism spectrum disorders, conduct disorder, oppositional defiant disorder, and substance use disorders (10). ADHD has been linked to significant decreases in quality of life and functioning, and has been found to be associated with higher risks of school failure, parental and family conflict, social rejection by peers, low self-esteem, and delinquent behavior in children and adolescents, and with academic and vocational underachievement, reduced occupational functioning, obesity, emotional dysregulation, unemployment, and suicide attempts in adolescence and adulthood (11).

## 2 Matters of consideration

In a comprehensive meta-analysis involving 19 studies and over 55,000 participants, it was found that 5.9% of youths meet the diagnostic criteria for ADHD (6). Another extensive meta-analysis, which included approximately a quarter of a million youths across 135 studies, revealed no significant differences in ADHD prevalence between North America and other global regions such as Europe, Asia, Africa, South America, and Oceania. Notably, these rates have remained consistent over the past three decades (7). However, despite the global consistency in epidemiological rates, there are significant disparities in administrative rates of diagnosis.

It is evident that there is significant variation in administrative prevalence rates among continents, among countries as well as within countries, such as US states, and among different ethnic groups (12–20). It should be noted that no comprehensive literature could be identified for Asia beyond reports of administrative prevalence rates at the country level [e.g., (21–23)]. Studies conducted in various countries, primarily in the “global North,” have highlighted a noticeable increase in the administrative diagnosis of ADHD in recent years (9, 10, 24–27). Furthermore, recent studies have revealed a clear divergence in the administrative prevalence of ADHD diagnosis when examining specific subpopulations within 48 different studies. This prompts a critical ongoing debate concerning the drivers behind this rise, including whether it is due to genuine increases in ADHD frequency, improved detection practices, or diagnostic inflation (25, 28).

A meta-analysis of 25 studies, involving over eight million participants, revealed a distinctive pattern: children and adolescents who are relatively younger compared to their classmates are more likely of receiving an ADHD diagnosis (29). This finding is supported by other studies (30, 31) highlighting the relative age effect on ADHD diagnosis, showing an increased proportion of younger children diagnosed in more recent birth cohorts.

Regarding medication trends, there has been a clear and consistent increase in prevalence of ADHD medication use over the past two decades, particularly in high-income countries. However, there are significant variations in medication use between different regions. Comparative analyses of medication consumption rates across various countries and regions highlight these disparities, with North America having the highest ADHD medication consumption, followed by Oceania and Northern and Central Europe, and other regions exhibiting significantly lower medication consumption rates (23, 32–36).

The situation in the United States is particularly noteworthy, as statistics indicate that stimulant medication was administered to 8–12% of male and 5% of female school-aged children in 2014 (37). Interestingly, these rates vary significantly across different US states, influenced by factors such as family and cultural values, healthcare infrastructure, media reports, and federal variations in educational policies linked to demands for performance and achievement (38–41).

The rise in administrative prevalence and medication usage in Western countries raises questions about the underlying dynamics. Is the increase in administrative prevalence and medication usage due to a real rise in cases or are there other factors at play? Have the definitional boundaries of ADHD changed, leading to the observed rising rates of diagnosis and treatment? Are environmental factors, such as changes in schooling practices and media coverage, contributing to the upward trend in diagnosis and medication usage? Could the increased awareness among physicians and patients about the potential of ADHD be a factor? And more broadly, the question arises: is the increasing administrative prevalence of ADHD and the rising prevalence of ADHD medication use essentially a positive development or should it rather be a cause for concern?

Comprehensive and conclusive answers to the aforementioned questions are beyond the scope of this commentary. However, we assert that in order to approach the answers to these questions, a thorough understanding of both the construct ADHD and the misconceptions that sometimes exist in this context are necessary.

We contend that ADHD is not a natural (and even less a homogeneous and uniform) entity that unfolds within an individual and could be understood independently of societal and environmental factors. Instead, we postulate that ADHD as diagnosis can better be conceptualized as a valid, pragmatical and useful social construct. Viewing ADHD as a social or cultural construct, in our opinion, does not entail its etiological reduction to culture (42). In our perspective, ADHD is not caused by culture but is constructed through categorization and classification, influenced by societal factors. This is reflected in the history of operational diagnostic criteria, notably with the introduction of ‘Attention-Deficit Disorder with or without hyperactivity’ in DSM-III in 1980 (11, 43). Recognizing ADHD as a social construct entails the conceptualization of ADHD as a disorder and the decision when to pursue medication treatment should be based on the benefits or harms it brings to individuals. This consideration should take into account the extent of impairment in relation to societal demands, as well as potential negative consequences stemming from stigmatization effects, which may need to be weighed against each other. Additionally, ethical aspects regarding symptom alleviation versus neuro-enhancement should be discussed when considering the indication for medical treatment.

## 3 Discussion

ADHD was typically conceptualized as a distinct and categorical entity with clear and definable boundaries that distinguish between individuals with the disorder and without, as well as demarcating its separation from other disorders. This conceptualization posits that ADHD originates within the individual [for a critique see also: (44)]. However, the disorder is characterized by marked heterogeneity on clinical, etiological, and pathophysiological levels. Individuals diagnosed with ADHD differ in terms of their core symptom combinations, level of impairment, comorbidities, and demographic characteristics. Patients also exhibit significant variation in their symptom profiles, symptom trajectories, clinical outcomes, and biological and neuropsychological correlates. No single factor or combination of factors serves as a definitive and comprehensive foundation for the condition. Consequently, ADHD cannot be classified as a distinct causal condition or a uniform entity. Instead, ADHD is best conceptualized as the extreme end of a spectrum, with individuals on the ADHD spectrum differing from those without ADHD primarily in term of degree rather than in fundamental nature. The impact of short or tall stature, which varies depending on conditions in daily life, may be considered an illustrative example of this concept with great height being more common in Western countries than in Southeast Asia.

The construct of ADHD has faced challenges and criticisms, primarily stemming from the perception that its diagnosis is “subjective,” lacking objective criteria. Critics argue that disorders should correspond to natural kinds and categories be defined by objective criteria. However, the argument that diagnosis and treatment are only justified if disorders represent a natural kind (based on objective criteria), seems unfounded especially in the realm of mental disorders. The question rather is whether a diagnosis holds pragmatic and clinical utility. ADHD has been acknowledged as meeting the standard criteria for validity of a mental disorder outlined by Robins and Guze (45). These criteria include inter-individual agreement, predictive of future outcomes, external correlates, such as associated biological factors, comorbidities, functional impairment, and response to treatment. Thus, as emphasized by Karalunas & Nigg (46), it is possible for different observable features to cluster in informative ways without necessitating the assumption of correspondence to a true or natural kind.

The assessment of ADHD is significantly challenged by the criterion of impairment. While this criterion is crucial, it is important to acknowledge that impairment does not solely originate from the individual but rather emerges from the interaction between the individual and their environment. More specifically, it is influenced by the interplay between environmental demands and the individual’s capacity to meet or adapt to these demands. This highlights that ADHD cannot be comprehensively understood as a natural entity confined within the individual, but rather as a phenomenon that arises within the context of external demands. Moreover, the concept of impairment attributed to ADHD is not inherent but is rather socially constructed, context-dependent, and contingent upon the environment. If ADHD were conceptualized and taught as a social construct, embracing a holistic approach that avoids reducing individuals solely to ADHD, recognizing the profound implications of diagnosis on identity development, and rejecting a reductionist perspective that focuses on isolated symptoms or behaviors, while also critically considering societal factors such as the pressure to perform, it could be hypothesized that both the diagnosis and treatment of ADHD would undergo a transformative paradigm shift.

When considering the social context, it is important to also account for differences between children and adults. For instance, in children the degree of impairment is highly associated with performance evaluation in the tightly structured educational setting and the largely predetermined family framework. In contrast, adults usually engage in professional and private contexts, bearing a higher level of personal responsibility, choice and control while often receiving less social support. The presence of an excessively restrictive and demanding setting may emphasize individual differences in attention, inhibitory control, and self-regulation. Educational policies, coupled with mounting pressure for heightened achievement and performance, can therefore contribute to elevated diagnostic rates (39).

In addition to the benefits that a diagnosis can bring to individuals, it is important to consider potential negative consequences, particularly the consideration of stigma effects. While the naturalistic view has contributed to reducing stigma surrounding mental health, it can still unintentionally reinforce the idea that mental disorders are solely brain-based conditions. This view might inadvertently perpetuate the notion that individuals with mental health issues are somehow fundamentally different from those without such conditions. Data highlighting the negative outcomes associated with diagnosis include nationally representative survey data from the United States. These findings from the Early Childhood Longitudinal Survey (ECLS) indicate that teachers perceive students with ADHD to be less successful in reading and math compared to their peers without ADHD. Interestingly, these perceptions were more negative than what would be expected based on actual differences in test scores, suggesting that negative attitudes toward ADHD may influence teachers` evaluations of their students` academic abilities. Similarly, parents also tend to evaluate their children’s academic abilities more negatively if they have been diagnosed with ADHD, even when accounting for actual differences in test scores (47). In addition to external stigma, self-stigma is a relevant aspect to consider, especially among children and adolescents. This phenomenon can result in the formation of a negative self-perception, as individuals come to see themselves as fundamentally different from their peers due to the ADHD label assigned to them (48).

ADHD medication not only effectively addresses the core symptoms of ADHD but also enhances overall functioning and quality of life (49–51). Additionally, there is a growing body of evidence supporting the long-term benefits and safety of ADHD medication (52, 53). Consequently, one might initially perceive the increased prevalence of ADHD medication users as a positive development, as it indicates that more individuals with impairments are receiving the help they need. However, this trend may also be influenced by an expansion of diagnostic criteria over the last decades, e.g., DSM-I through DSM-5 (54). Furthermore, there is an ongoing debate regarding the use of pharmacological treatment for individuals with milder ADHD symptoms. This debate extends to whether “healthy” individuals should have access to ADHD medication to boost their performance. In both scenarios, the central considerations (again) revolve around utility and functionality. It is crucial to carefully weigh the negative effects, such as potential side effects and experiences of stigma, against the positive outcomes, including improved overall functioning, performance and quality of life rather than merely reduction of symptoms (55). In complex cases with major psychiatric comorbidities (e.g., affective disorders) and additional medication (e.g., SSRIs), an even greater emphasis needs to be laid on potential adverse events (56).

Ethical concerns, such as issues related to competitiveness and fairness, as well as the impact of detrimental, “unhealthy” performance pressure, must also be thoroughly addressed. In future discussions and debates on this topic, it is important to keep these factors as a central focus, all the while considering how the concept of ADHD can be understood, as well as what it is not.

In summary, we think that ADHD should not be viewed as a natural homogeneous entity within an individual, independent of societal and environmental influences. Instead, it is more appropriately conceptualized as a social construct because the (varying) diagnostic criteria (agreed upon by experts based on evidence) define who will fall within this category, assessment procedures and clinical practice systems will determine who will receive a diagnosis, and the diagnosis (and consequences) will at least potentially alter the self-concepts of those diagnosed (i.e., may lead to (self-)stigmatization or de-stigmatization). The model presented here corresponds with the recent debate as to whether the application of the concept of neurodiversity to neurodevelopmental disorders could lead to the development of new approaches to therapeutic interventions for people with ADHD or autism (57). In contrast to the previous “disorder model”, in which therapy is always primarily aimed at eliminating the core deficits of the disorder, the primary therapeutic target according to the neurodiversity concept would be to change the context, which should be designed in such a way that neurodiverse people can make positive and affirmative experiences and draw on their resources and specific characteristics (58).

To sum things up, there is no “true” ADHD against which the diagnostic criteria could be verified or falsified, and there are no purely natural courses of individuals with ADHD that develop outside a specific societal-cultural context. This does imply that a notion such as “ADHD does not exist” does not make sense but also that “objective” diagnostic biomarkers are by no means necessary or sufficient to validate the diagnostic category of ADHD.

## Author contributions

TB: Conceptualization, Writing – original draft, Writing – review & editing. AH: Writing – original draft, Writing – review & editing. SH: Writing – original draft, Writing – review & editing. KM: Writing – original draft, Writing – review & editing.
